# Cultural influences on face scanning are consistent across infancy and adulthood

**DOI:** 10.1016/j.infbeh.2020.101503

**Published:** 2020-11

**Authors:** Jennifer X. Haensel, Mitsuhiko Ishikawa, Shoji Itakura, Tim J. Smith, Atsushi Senju

**Affiliations:** a Department of Psychological Sciences, Birkbeck, University of London, Malet Street, London, WC1E 7HX, United Kingdom; b Department of Psychology, Graduate School of Letters, Kyoto University, Yoshida-honmachi, Sakyo-ku, Kyoto, 606-8501, Japan; c Center for Baby Science, Doshisha University, 4-1-1 Kizugawadai, Kizugawa, Kyoto, 619-0225, Japan

**Keywords:** Face perception, Cultural differences, Dynamic faces, Eye tracking, Face scanning, Social development

## Abstract

The emergence of cultural differences in face scanning is thought to be shaped by social experience. However, previous studies mainly investigated eye movements of adults and little is known about early development. The current study recorded eye movements of British and Japanese infants (aged 10 and 16 months) and adults, who were presented with static and dynamic faces on screen. Cultural differences were observed across all age groups, with British participants exhibiting more mouth scanning, and Japanese individuals showing increased central face (nose) scanning for dynamic stimuli. Age-related influences independent of culture were also revealed, with a shift from eye to mouth scanning between 10 and 16 months, while adults distributed their gaze more flexibly. Against our prediction, no age-related increases in cultural differences were observed, suggesting the possibility that cultural differences are largely manifest by 10 months of age. Overall, the findings suggest that individuals adopt visual strategies in line with their cultural background from early in infancy, pointing to the development of a highly adaptive face processing system that is shaped by early sociocultural experience.

## Highlights

• We compare face scanning patterns in British and Japanese infants and adults.
• We investigate whether cultural differences become more distinct with age.
• Age and culture independently modulated scanning of static and dynamic faces.
• Cultural differences in face scanning emerge within the first year of life.
• Scanning strategies are in line with cultural background and stage in development.

## 1 Introduction

The human face represents an important visual stimulus in our everyday life, allowing us to identify others, infer emotional states, and participate in shared attention (Bruce & Young, 1998; Haxby, Hoffman, & Gobbini, 2000; Hoffman & Haxby, 2000). Over the course of the first year of life, recognition abilities become increasingly optimised for faces of a shared ethnic background, but not for faces of less familiar ethnicities (Anzures, Quinn, Pascalis, Slater, & Lee, 2013; Anzures, Pascalis, Quinn, Slater, & Lee, 2011; Kelly et al., 2007, 2009). This process of perceptual narrowing reflects an adaptive mechanism for fine-tuning to socially relevant information (Nelson, 2001), challenging the notion that processes underlying face perception are universal and highlighting the role of postnatal social experience in the development of expert face processing.

More recently, eye tracking studies with adults have revealed cultural differences in visual strategies during face perception tasks. In recognition tasks for faces with neutral expressions, Western Caucasian (WC) participants exhibited greater scanning of the eyes and mouth than East Asian (EA) participants, whereas EAs showed more fixations on the nose than WCs (Blais, Jack, Scheepers, Fiset, & Caldara, 2008; Kelly et al., 2010; Kelly, Liu et al., 2011; Kita et al., 2010; Rodger, Kelly, Blais, & Caldara, 2010). This has been suggested to reflect the culture-typical patterns of analytic (WC) versus holistic (EA) allocation of attention (Blais et al., 2008; Chua, Boland, & Nisbett, 2005; Masuda & Nisbett, 2006), whereby WC individuals tended to extract focal information and EAs used their extrafoveal vision more effectively, thereby allowing them to extract key visual information by fixating the nose (Caldara, 2017; Miellet, Vizioli, He, Zhou, & Caldara, 2013; Miellet, He, Zhou, Lao, & Caldara, 2012). When viewing emotionally expressive face stimuli, WCs showed more mouth looking than EAs, while EAs exhibited increased scanning of the eye region compared to WCs (Jack, Caldara, & Schyns, 2012; Jack, Blais, Scheepers, Schyns, & Caldara, 2009; Senju, Vernetti, Kikuchi, Akechi, & Hasegawa, 2013; Senju, Vernetti, Kikuchi, Akechi, Hasegawa, Johnson, 2013). This could possibly reflect an adaptation to how cultures differentially use facial features to express emotions, with computational modelling having demonstrated that East Asians represented the intensity of emotional expressions with movements of the eyes, whereas Western Caucasians used other face regions including the mouth (Jack et al., 2012). More recently, this scanning pattern has also been replicated within a live dyadic social interaction paradigm, showing that cultural differences can also be observed beyond screen-based studies that employ static or dynamic stimuli (Haensel et al., 2020).

Although cultural modulations on face scanning have been reported across different face processing tasks, current evidence is largely restricted to data from adult populations. Using static stimuli depicting faces with neutral expressions, Kelly, Liu et al. (2011) studied the developmental trajectory for scanning strategies of British and Chinese 7- to 12-year-olds, and found that viewing patterns corresponded with those of adults from the respective culture (central (nose) scanning in Chinese participants, and more distributed, triangular-like scanning (eyes and mouth) in British participants). To our knowledge, however, only two cross-cultural studies on face scanning have been conducted with infants or very young children, with findings showing that cultural differences in face scanning can be observed in 7-month-old infants (Geangu et al., 2016) and children aged 1–7 years (Senju, Vernetti, Kikuchi, Akechi, Hasegawa, 2013). When free-viewing static, emotionally expressive face images, 7-month-old British and Japanese infants exhibited scanning patterns consistent with those of adults: compared to the British group, Japanese infants showed less mouth scanning and more fixations on the eye region (Geangu et al., 2016), which may reflect early culture-specific learning of the visually informative face regions (cf., Jack et al., 2012). Similar patterns were also found for British and Japanese children aged between 1 and 7 years when free-viewing dynamic, emotionally expressive face stimuli (Senju, Vernetti, Kikuchi, Akechi, Hasegawa, 2013). However, given that only a single age group was tested, little is currently known about the developmental trajectory for face scanning. To address this question, cross-sectional or longitudinal designs are required.

Beyond cross-cultural research, several cross-sectional studies have previously investigated age-related changes in face scanning within a single cultural group of WC infants. For instance, when presented with static images of emotionally expressive faces, older infants (12 months) showed more upper face looking (including the eyes) than younger infants (5 months; Miguel, McCormick, Westerlund, & Nelson, 2019), and adults further looked more at the eye region than infants aged 4 and 7 months (Hunnius, de Wit, Vrins, & von Hofsten, 2011). In later infancy beyond the first year of life – coinciding with the age range when infants enter the word acquisition stage – a shift from eye to mouth looking has also been reported, especially for talking faces (Frank, Vul, & Saxe, 2012; Król, 2018). This pattern could reflect adaptive mechanisms for learning requirements at each age, with infants in the first year of life benefiting from eye looking for social learning and early non-verbal communication (Csibra & Gergely, 2006; Kleinke, 1986); for instance, eye contact can allow infants to engage in subsequent gaze following and joint attention (Scaife & Bruner, 1975; Senju & Csibra, 2008). During the specific period in development when infants enter the word acquisition stage (in the second year of life; Oller, 2000), an increased focus on the moving mouth may provide a source for language learning (Hillairet de Boisferon, Tift, Minar, & Lewkowicz, 2018). Although the underlying mechanisms require further examination, such findings support the notion that developmental changes in face scanning occur within and beyond the first year of life. It remains unclear, however, whether developmental trajectories for scanning patterns differ between cultural groups. Several studies also examined developmental changes in scanning patterns of infants (from a single cultural group) when presented with faces of their own versus an unfamiliar ethnicity displaying neutral expressions during muted speech (Liu et al., 2011; Wheeler et al., 2011; Xiao, Xiao, Quinn, Anzures, & Lee, 2013). Unlike the findings from cross-cultural studies (Geangu et al., 2016; Senju, Vernetti, Kikuchi, Akechi, Hasegawa, 2013), scanning patterns were dependent on the ethnicity of the face stimuli. For instance, with increasing age, 6- to 10-month-old WC infants looked longer at the eyes and less at the mouth of faces from their own ethnicity compared to faces of Chinese ethnicity (Wheeler et al., 2011; Xiao et al., 2013). The role of ethnicity in modulating face scanning, however, remains unclear since cross-cultural studies found no such support (Geangu et al., 2016; Senju, Vernetti, Kikuchi, Akechi, Hasegawa, 2013). Additionally, scanning patterns were examined in only a single cultural group, such that developmental trajectories in face scanning for different cultures remain unknown.

Adopting a developmental framework will help identify the time course of emerging cultural differences in face scanning, which would reflect increasing adaptations to the postnatal cultural environment. This in turn offers insight into possible mechanisms that can ultimately explain how postnatal social experience modulates scanning strategies, and furthermore point to the potential functional significance of these cultural differences at specific age ranges in early infancy. To address the gaps in the literature, the current cross-sectional investigation examined face scanning patterns of British and Japanese infants (aged 10 and 16 months) and adults to compare developmental trajectories and establish any cultural differences within a single study.

Given that early cultural differences have previously been observed by the end of the first year of life, we examined face scanning in 10-month-old infants. Although the examination of age-related changes in face scanning irrespective of cultural background was secondary to our objectives, it was expected that early cultural differences should be present at 10 months. However, evidence is currently limited, and it is possible that cultural differences could emerge later in development. An older infant age group consisting of 16-month-olds was therefore also included. From a developmental perspective, cultural differences should become more distinct in 16-month-olds than in 10-months-olds, and even more so in adults than in infants as individuals become increasingly adapted to their cultural environment with age.

Given that face stimulus characteristics may affect scanning strategies and the existing evidence for infants and young children is limited to dynamic, emotionally expressive face stimuli (Geangu et al., 2016; Senju, Vernetti, Kikuchi, Akechi, Hasegawa, 2013), it also remains unclear to what extent current findings can be observed for non-expressive faces. Face stimuli in the current study were presented in three different conditions to examine viewing patterns in a more comprehensive manner: *static* with neutral expression, as commonly employed in cross-cultural face scanning studies with adults (Blais et al., 2008); *dynamic-neutral*, showing a dynamic face with neutral expression, as in previous infant face scanning studies examining perceptual narrowing (Liu et al., 2011; Wheeler et al., 2011); and *dynamic-expressive*, displaying a dynamic, emotionally expressive face to consider findings showing divergent scanning patterns for emotionally expressive versus neutral faces. In line with findings based on adult studies employing face stimuli with neutral expressions (e.g., Blais et al., 2008), it was expected that British participants would exhibit greater triangular scanning (eyes and mouth) in the static and dynamic-neutral conditions than Japanese individuals; Japanese participants, meanwhile, were predicted to show more central (nose) face looking than British individuals. However, given the task-dependent nature of these earlier studies, we acknowledged the possibility that the previously reported triangular versus central scanning patterns could also reflect culture-typical strategies for effective face recognition or face categorisation. Given that the present study employed a free-viewing paradigm, infants and adults may therefore exhibit different scanning patterns. In line with studies employing emotionally expressive face stimuli to examine face scanning in adults and children (Jack et al., 2009; Senju, Vernetti, Kikuchi, Akechi, Hasegawa, 2013), it was expected that Japanese participants would look more at the eye region of dynamic-expressive faces than the British group; British participants, meanwhile, were expected to show greater mouth looking. To make the present analysis comparable with previous cross-cultural studies that are each limited to a particular face stimulus type (i.e., static, dynamic-neutral, dynamic-expressive), we did not directly compare between stimulus types, but instead examined cultural differences and age-related changes for each type separately. The presented stimuli furthermore included faces of both White-British and Japanese ethnicity to account for a possible role of face ethnicity in modulating scanning behaviour (Fu, Hu, Wang, Quinn, & Lee, 2012; Liu et al., 2011; Wheeler et al., 2011; Xiao et al., 2013), although it was acknowledged that such ethnicity effects are not consistent across studies (Geangu et al., 2016; Senju, Vernetti, Kikuchi, Akechi, Hasegawa, Johnson, 2013).

## 2 Materials and methods

### 2.1 Participants

The study was conducted in the UK (Birkbeck, University of London) and in Japan (Kyoto University), with each cultural group consisting of 10- and 16-month-olds, and adults. The study was approved by the psychology ethics committee of Birkbeck, University of London and Kyoto University, and was conducted in accordance to the Declaration of Helsinki. Adult participants and families were recruited via internal university databases. Adult participants and parents/guardians provided written informed consent prior to the study.

British participants were born and raised in the UK (except one 10-month-old born in Germany), were of White ethnicity, had never lived outside Western Europe/USA/Canada, and indicated English as their native language (adults) or the caregiver communicated in English (infants). Japanese participants were born and raised in Japan, were of Japanese ethnicity (except one 16-month-old whose secondary caregiver was of White ethnicity), had never lived outside East Asia, and indicated Japanese as their first language (adults) or the caregiver communicated in Japanese (infants). Most infants were of middle socioeconomic status background. All participants, or the caregivers for infants, reported normal or corrected-to-normal vision and hearing, and no developmental conditions.

Additional participant information is provided in Table 1. Sample sizes (Table 1) were identified based on previous studies (Liu et al., 2011; Xiao et al., 2013). Fourteen additional British infants were tested but excluded from analysis due to low-quality data in the form of flicker (*N* = 9; this also resulted in not triggering the gaze-contingent central fixation point that preceded a face trial without manual key-press from the experimenter – see procedure below), equipment failure (*N =* 1), fussiness (*N =* 2), failed calibration (*N =* 1), or not meeting ethnicity requirements (*N =* 1). Two British adults were excluded due to flicker or equipment failure. In Japan, five infants were removed from analysis due to fussiness, and three adults because of flicker.

**Table 1:** Participant characteristics by cultural group and age.

Age group	Cultural group	N (female)	Mean age (range)
10 months	British	26 (10)	307 days (288−330 days)
	Japanese	22 (11)	306 days (289−323 days)

16 months	British	26 (11)	474 days (446−507 days)
	Japanese	15 (5)	481 days (449−534 days)

Adults	British	31 (16)	27.35 years (19−40 years)
	Japanese	30 (17)	21.73 years (18−31 years)

The visit typically lasted 45 min for infants, which included play time at the beginning to familiarise infants with the testing environment, and 30 min for adults. The eye tracking experiment lasted between 10 and 12 min. In line with departmental ethics guidelines for each institution, British families were reimbursed travel expenses and received a T-Shirt and certificate of participation, and Japanese families were reimbursed ¥3000 for their time. Adult participants received £8 (UK) or ¥1000 (Japan) for their time.

### 2.2 Apparatus

Eye movements were recorded using a Tobii TX300 eye tracker (Tobii Technology, Sweden) at 120 Hz sampling rate. Although the TX300 eye tracker can run at a maximum sampling rate of 300 Hz, a lower rate was chosen to improve the quality of data collected from infants (Saez de Urabain, Johnson, & Smith, 2015). All stimuli were presented on a 23″ monitor, and the experimental protocol was controlled through MatLab (R2013a, MathWorks) using the Psychophysics toolbox (Version 3). Two external speakers, each located next to one side of the monitor, were used to play sounds. Participants were monitored via a built-in webcam.

### 2.3 Procedure

Participants were welcomed in the reception room where the experimenter explained the study, collected written informed consent, and asked caregivers/adult participants to fill in a demographic questionnaire. Participants were then guided to the testing room and sat on a chair (adults) or the caregiver’s lap (infants) at approximately 65 cm distance from the screen. An infant-friendly video was presented, with the tracked gaze and head locations visualised on the screen to facilitate accurate positioning of the eye tracker. Participants then completed a five-point calibration procedure (caregivers were asked to close their eyes). To keep infants’ visual attention on-screen, each calibration point was presented as a colourful, inward-turning spiral that was accompanied by an attention-grabbing sound. Gaze data for each eye was visualised on the laptop of the experimenter. Calibration was repeated when gaze data was not available for two or more points, in which case a second or third calibration attempt was conducted before starting the study protocol.

The protocol involved free-viewing face stimuli, and also two cognitive tasks that addressed a research question unrelated to the current study and therefore are not reported here. These cognitive tasks were presented between the three blocks of face scanning trials. Additionally, a colourful, inward-turning spiral (used for calibration) was presented between each experimental trial (i.e., each face) and block (i.e., face stimuli belonging to the same stimulus type) to examine possible age or cultural group differences in spatial accuracy (see Supplementary Materials for further details). No significant group differences in accuracy were found.

**Fig. 1:**
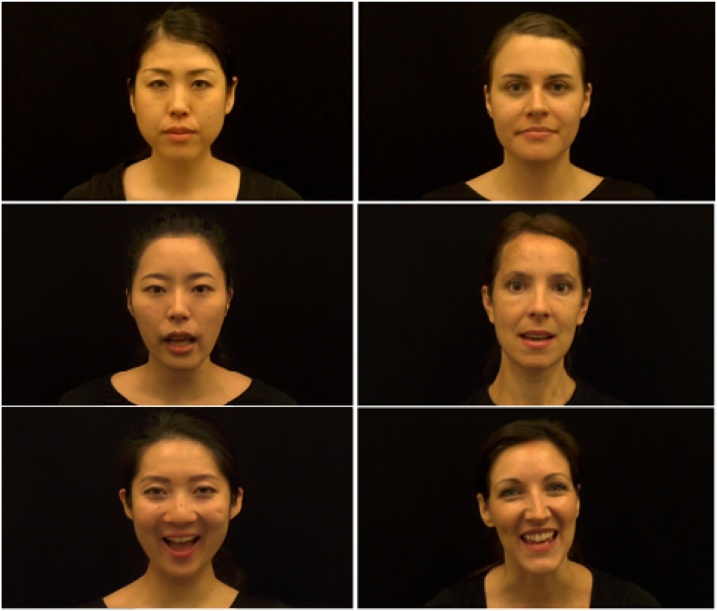
Example screenshots in the static (top), dynamic-neutral (middle), and dynamic-expressive condition (bottom).

Each face scanning trial was preceded by a gaze-contingent fixation point located in the centre of the screen to ensure that the trial could only be triggered when participants were visually attending to the screen. Once the trial began, a single face was presented for 18 s. The static condition displayed an image of a face with neutral expression; dynamic-neutral faces showed actors articulating the syllables *do re mi fa sol la ti do* (which commonly exist in both English and Japanese language, and would therefore minimise language-specific mouth movements); dynamic-expressive faces presented smiling actors who articulated the syllable sequence and simultaneously performed one of two facial actions (peekaboo, or head nodding; Fig. 1). Periods during which the face was occluded in the peekaboo action were excluded from analysis. All face scanning trials were presented with unsynchronised instrumental music, and the original sound (in the two dynamic conditions) was muted. Timings of facial actions were matched across actors by training them to perform the action sequences in time to a metronome that played at 60 beats per minute; this was possible since the original sounds were muted in the video editing process.

Three face scanning blocks were presented, with the first, second, and third block consisting of static, dynamic-neutral, and dynamic-expressive face stimuli, respectively. The order of the blocks was fixed after pilot studies indicated that more engaging social and dynamic stimuli at the end of the protocol ensured infants’ visual attention remained on-screen. In each of the three blocks, participants were presented with faces of four actors (two of White-British and two of Japanese ethnicity). In total, each participant therefore viewed twelve faces, with this number of trials in line with previous cultural face scanning studies with infants (Liu et al., 2011; Wheeler et al., 2011; Xiao et al., 2013). The order of ethnicity and the order of actors were counterbalanced across participants and groups, and faces were never repeated (i.e., each participant was shown twelve different faces). All actors were female, aged between 25 and 35, and had dark brown or black hair. They wore black T-Shirts, had no jewellery, glasses, or visible make-up, the hair was tied back, and faces were shown in frontal view against a black background while making eye contact with the camera. Stimuli were edited in Final Cut Pro X (Version 10.0.8) to display faces measuring 16.5° (height) x 12.0° (width), controlled for luminance, and in colour at 1920 × 1080 resolution. All stimuli were aligned at the midpoint between the nose tip and bridge.

When an infant became inattentive, an auditory attention grabber was triggered by the experimenter who monitored participants via a webcam. In the case that an infant became fussy, the protocol was interrupted to allow for a short play break and resumed after an additional five-point calibration procedure, or the study was stopped.

### 2.4 Data pre-processing and design

Data loss (including blinks and attention off-screen) was lower for British adults (*M =* 18.15 %; *SD =* 8.77 %) than for Japanese adults (*M =* 26.65 %; *SD =* 10.01 %), and lower for Japanese infants than for British infants (Japanese 10-month-olds: *M =* 38.16 %, *SD* = 11.65 %; British 10-month-olds: *M =* 55.54 %, *SD* = 12.07 %; Japanese 16-month-olds: *M =* 37.43 %, *SD* = 11.23 %; British 16-month-olds: *M =* 45.68 %, *SD* = 14.18 %). This was largely as a result of infants visually orienting away from the screen during a face trial before the experimenter used an attention-grabber and the infant re-oriented toward the screen. For the face scanning analysis, fixation time on different facial features (eyes, nose, and mouth) was calculated proportional to overall face fixation time rather than trial duration (see below for details). Furthermore, raw gaze data was smoothed, interpolated, and subsequently parsed into fixations using the semi-automatic GraFIX software (Saez de Urabain et al., 2015) that was specifically developed for fixation coding with data of varying quality, which is common in infancy and also across ethnicities (Blignaut & Wium, 2014). GraFIX was chosen due to its two-step approach involving rapid automatic pre-processing to smooth and interpolate gaze data, followed by an optional moderation stage for fixation coding, which allows the user to manually flag, delete, or modify fixations that were judged to be incorrectly detected by the automatic algorithm. The input parameter values for initial automatic processing, the guidelines used for manual moderation of automatically coded fixations, and the details on agreements for second-coding are all provided in the Supplementary Materials. A minimum fixation duration of 100 msec was used.

**Fig. 2:**
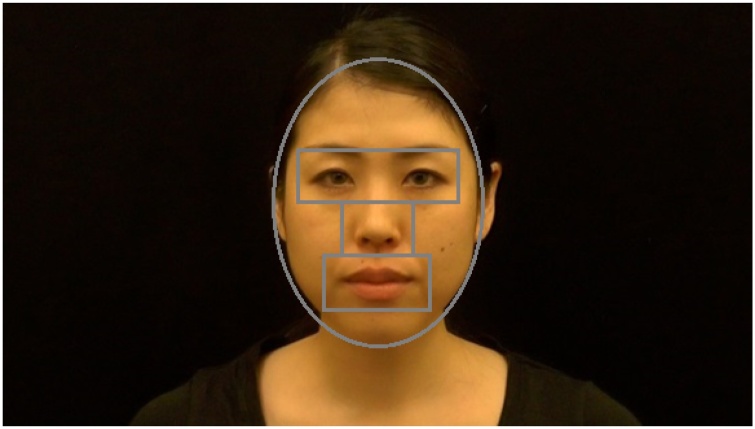
Regions-of-interest superimposed onto a face.

Regions-of-interest (ROIs) included the eyes, nose, and mouth (Fig. 2). ROIs were dynamic to account for blocks that presented moving faces, and regions were manually coded in MatLab. To examine age-related changes in scanning behaviour of British and Japanese individuals, Generalized Estimating Equations (GEE) were conducted. The GEE method was chosen since – unlike traditional repeated-measures analysis of variance (ANOVA) approaches – it does not assume independence for repeated-measures factors. For the present analyses, linear GEE models were estimated with an identity link, an unstructured correlation matrix, and a robust estimator.

The between-subject factors Culture (British/Japanese) and Age (10 months/16 months/adults) as well as the within-subject factor ROI (eyes/nose/mouth) were entered into the model. In the initial analysis, the factor Face Ethnicity (White-British/Japanese) was also included, but no significant main effect or interactions with Face Ethnicity could be observed (all *p* > 0.05). Face scanning was therefore examined by collapsing across the two levels of the Face Ethnicity factor. Cumulative fixation time in each ROI was calculated proportional to face fixation time (Fig. 2), and represented the dependent variable after logit transformation, which is effective for proportion data with many values close to the boundary of 0 and 1 (zero-entries were replaced with a small error term ε = 0.001). As discussed earlier, scanning behaviour was examined separately for each stimulus type (static, dynamic-neutral, dynamic-expressive), which also allowed for simpler models. In the following, face scanning findings are therefore presented separately for static, dynamic-neutral, and dynamic-expressive faces.

## 3 Results

### 3.1 Static faces

A significant main effect of ROI was revealed (Wald χ^2^_(2)_ = 274.84, *p* < 0.001), suggesting that scanning was not homogeneous across facial features, with the eyes being scanned more than the nose or mouth. An effect of Age was also revealed (Wald χ^2^_(2)_ = 14.02, *p* = 0.001), indicating age-related differences in cumulative scanning time of the core facial features (eyes, nose, mouth): 10-month-olds scanned the features most, followed by the 16-month group, and the adults. The effects of Culture (Wald χ^2^_(1)_ = 0.03, *p =* 0.601) and Age *x* Culture (Wald χ^2^_(2)_ = 4.42, *p* = 0.110) were not significant. Crucially, the relevant interactions to investigate group differences in face scanning are those involving the factor ROI; these examine whether overall fixation time on different ROIs was modulated by culture and/or age. A significant ROI *x* Culture interaction was revealed (Wald χ^2^_(2)_ = 6.98, *p* = 0.031), indicating that face scanning differed between cultural groups. Face scanning also differed between ages (ROI *x* Age: Wald χ^2^_(4)_ = 18.78, *p =* 0.001). However, the ROI *x* Age *x* Culture interaction was not significant (Wald χ^2^_(4)_ = 0.744, *p* = 0.946), thereby not supporting the prediction that cultural differences would become more distinct with age when viewing static faces.

**Fig. 3:**
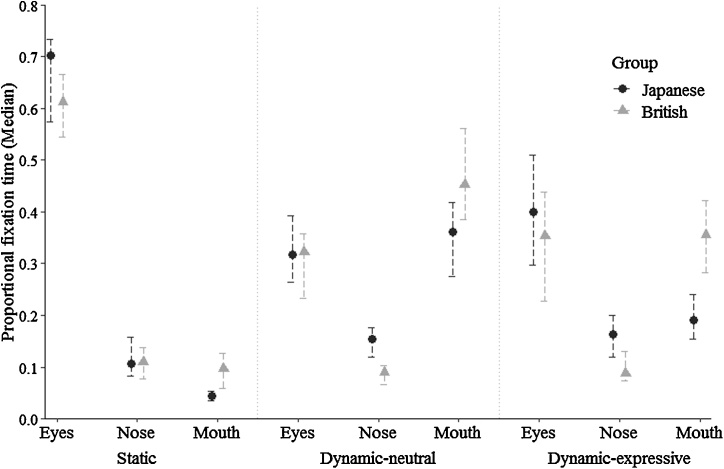
Median proportional fixation times for each ROI, stimulus type, and cultural group. Error bars represent 95 % CI for the median.

The ROI *x* Culture and ROI *x* Age interactions were followed up separately at each level of ROI to assess cultural and age-related differences in eye, nose, and mouth scanning (Bonferroni-corrected). Follow-up analyses were conducted using Mann Whitney *U* Tests using untransformed data for more intuitive interpretation of the results. These revealed that British participants exhibited more mouth scanning than the Japanese group (*U* = 1913, *p* = 0.001, *r =* 0.268), but significant cultural differences were not observed for scanning of the eyes (*U* = 2384, *p* = 0.134, *r =* 0.122) or nose (*U* = 2583, *p* = 0.455, *r =* 0.061; Fig. 3).

**Fig. 4:**
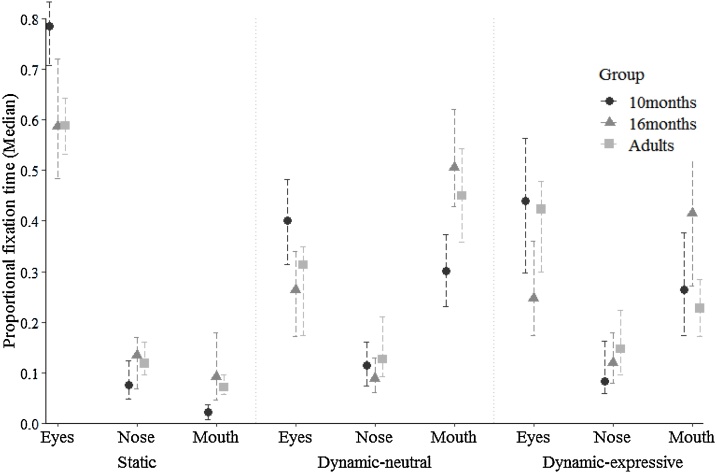
Median proportional fixation times for each ROI, stimulus type, and age group. Error bars 95 % CI for the median.

With respect to age differences (Fig. 4), 10-month-olds exhibited more eye scanning than 16-month-olds (*U* = 603, *p* = 0.002, *r =* 0.332) and adults (*U* = 817, *p* < 0.001, *r =* 0.378; no significant difference between 16-month-olds and adults: *U* = 1249, *p* = 0.992, *r =* 0.009). For the nose region, adults exhibited significantly more scanning than 10-month-olds (*U* = 1046, *p* = 0.011, *r =* 0.244). Although the 16-month group showed the highest median for proportional fixation time (see Fig. 4), no significant age differences were observed, possibly given greater variability in the data (10 versus 16 months: *U* = 838, *p* = 0.229, *r =* 0.127; 16 months versus adults: *U* = 1104, *p* = 0.317, *r =* 0.099). Finally, the 10-month-group showed significantly less mouth scanning than 16-month-olds (*U* = 520, *p* < 0.001, *r =* 0.406) and adults (*U* = 801.50, *p* < 0.001, *r =* 0.388) no significant difference between 16-month-olds and adults: *U* = 1102, *p* = 0.311, *r =* 0.100).

### 3.2 Dynamic-neutral faces

As with static faces, a main effect was observed for ROI (Wald χ^2^_(2)_ = 122.05, *p* < 0.001), with the mouth being scanned most, followed by the eye region, while the nose was scanned least. A main effect was also revealed for Culture (Wald χ^2^_(1)_ = 15.10, *p* < 0.001), with British participants scanning the features more than the Japanese group, but not for Age (Wald χ^2^_(2)_ = 5.48, *p =* 0.065). The Age *x* Culture interaction was also significant (Wald χ^2^_(2)_ = 25.80, *p* < 0.001), with Bonferroni-corrected follow-up analyses indicating that British (but not Japanese) adults fixated the ROIs more than British 10-month-olds (*U =* 228.50, *p* = 0.005). Note that this cannot be explained by age differences in data loss since fixation times were calculated proportional to face looking time. No other significant age or cultural differences were observed (all *p* > 0.025, Bonferroni-corrected).

The findings on group differences in face scanning were consistent with those observed for static stimuli. Face scanning significantly differed between cultural groups (ROI *x* Culture: Wald χ^2^_(2)_ = 18.05, *p* < 0.001) and age groups (ROI *x* Age: Wald χ^2^_(4)_ = 24.25, *p* < 0.001), but the hypothesis concerning age-related changes in the magnitude of cultural differences in scanning behaviour was not supported (ROI *x* Age *x* Culture: Wald χ^2^_(4)_ = 7.92, *p* = 0.095).

Bonferroni-corrected follow-up analyses of the significant ROI *x* Culture interaction revealed, as with static faces, more mouth scanning in British compared to Japanese participants (*U =* 2124, *p* = 0.013, *r =* 0.203). As with findings for static faces, cultural differences in eye scanning were not observed (*U =* 2598, *p* = 0.490, *r =* 0.056). In contrast to the findings for static faces, the Japanese group exhibited more nose scanning than British participants (*U =* 1670, *p* < 0.001, *r =* 0.342; Fig. 3).

Bonferroni-corrected post-hoc analyses of age differences in face scanning furthermore revealed – as with static face stimuli – significantly greater eye scanning in the 10-month group compared to both the 16-month-olds (*U =* 592, *p* = 0.001, *r =* 0.342) and adults (*U =* 1002.50, *p* = 0.005, *r =* 0.270; no significant difference between 16-month and adults: *U =* 1143.50, *p* = 0.465, *r =* 0.072; Fig. 4). As with the static condition, adults and 16-month-olds exhibited greater mouth scanning than the 10-month group (adults versus 10 months: *U =* 1049, *p* = 0.011, *r =* 0.243; 16 vs 10 months: *U =* 498, *p* < 0.001, *r =* 0.424; no significant difference between 16 months and adults: *U =* 975, *p* = 0.060, *r =* 0.186). Finally, nose scanning did not significantly differ between the two infant groups (*U =* 865, *p* = 0.327, *r =* 0.104); in contrast to the findings for static stimuli, however, adults showed more nose scanning than 16-month-olds (*U =* 840, *p* = 0.005, *r =* 0.277), and no significant differences were observed compared to 10-month-olds (*U =* 1115, *p* = 0.033, *r =* 0.204).

### 3.3 Dynamic-expressive faces

A main effect was observed for ROI (Wald χ^2^_(2)_ = 65.46, *p* < 0.001), with the eye and mouth region showing similar proportional looking times while the nose was scanned the least. A main effect of Age was also observed (Wald χ^2^_(2)_ = 10.26, *p* = 0.006) – 16-month-olds scanned the facial features most – but not for Culture (Wald χ^2^_(1)_ = 2.28, *p* < 0.131). The Age *x* Culture interaction was significant (Wald χ^2^_(2)_ = 6.90, *p* = 0.032) and Bonferroni-corrected follow-up analyses indicated that British (but not Japanese) 10-month-olds fixated ROIs less than British adults (*U =* 163, *p* < 0.001) and British 16-month-olds (*U =* 182, *p* = 0.004). Additionally, British adults looked more at ROIs than Japanese adults (*U =* 286, *p* = 0.010). No other group differences in proportional scanning time for ROIs were observed (all *p* > 0.025, Bonferroni-corrected).

The findings on face scanning mirrored those obtained for both static and dynamic-neutral face stimuli. As before, scanning patterns differed between cultural groups (ROI *x* Culture: Wald χ^2^_(2)_ = 17.86, *p* < 0.001) and age groups (ROI *x* Age: Wald χ^2^_(4)_ = 18.48, *p* = 0.001), but the hypothesised developmental changes in cultural differences were not supported (ROI *x* Age *x* Culture: Wald χ^2^_(4)_ = 3.46, *p* = 0.484).

Non-parametric follow-up analyses (Bonferroni-corrected) revealed that cultural differences in face scanning mirrored those observed for dynamic-neutral stimuli. While no significant differences were found for eye scanning (*U =* 2294, *p* = 0.066, *r =* 0.150), Japanese participants engaged in greater nose (*U =* 1939, *p* = 0.001, *r =* 0.260) and less mouth scanning (*U =* 1664, *p* < 0.001, *r =* 0.345) than the British group (Fig. 3).

With respect to age differences, post-hoc comparisons showed patterns of findings that were consistent with those obtained for static and dynamic-neutral conditions. Sixteen-month-olds engaged in less eye scanning compared to the 10-month group (*U =* 663, *p* = 0.008, *r =* 0.280) and to adults (*U =* 897, *p* = 0.016, *r =* 0.239; no significant difference between adults and 10-month-olds: *U =* 1397, *p* = 0.683, *r =* 0.039; Fig. 4). Adults furthermore engaged in greater nose scanning than the 10-month group (*U =* 1022, *p* = 0.007, *r =* 0.258; no significant differences between 16 months and adults: *U =* 996, *p* = 0.082, *r =* 0.172, and 16 versus 10 months: *U =* 854, *p* = 0.285, *r =* 0.113). Finally, mouth scanning was greater in 16-month-olds compared to both the 10-month-olds (*U =* 606, *p* = 0.002, *r =* 0.330) and adults (*U =* 740, *p* < 0.001, *r =* 0.345; no significant difference between adults and 10-month-olds: *U =* 1368, *p* = 0.558, *r =* 0.056).

### 3.4 Summary of ROI findings

For all three stimulus types – static, dynamic-neutral, dynamic-expressive – British participants engaged in more mouth scanning than the Japanese group. For dynamic-neutral and dynamic-expressive faces, Japanese individuals furthermore exhibited more nose scanning than British individuals. Against predictions, significant cultural differences were not observed for eye scanning in any of the face stimulus types used in the current study, thereby not supporting the greater triangular (eyes and mouth) scanning for static and dynamic-neutral faces in British compared to Japanese participants, and not supporting increased eye looking for dynamic-expressive faces in Japanese compared to British participants. Furthermore, 10-month-olds showed greater eye scanning and less mouth scanning compared to the two older age groups. Mouth scanning tended to be greatest at 16 months, while nose scanning was highest in the adult group. Together, the findings from the ROI analysis suggest that although cultural differences in face scanning as well as (culture-independent) age-related changes in face scanning were observed consistently across different stimulus types, the pattern of results did not support the prediction that cultural differences become significantly more distinct with age.

## 4 Discussion

This cross-sectional study aimed to explore the developmental emergence of cultural differences in face scanning, by contrasting British and Japanese 10- and 16-month-olds as well as adults. Crucially, the findings point to independent effects of culture and age, but no interaction between these two factors. This suggests that cultural differences in face scanning – at least those observed in the current experimental paradigm – were largely established by 10 months.

The precise manifestation of observed cultural differences only partially replicated previous studies with older children and adults. British participants exhibited greater mouth looking than Japanese individuals across all stimulus types, pointing to a consistent marker for cultural differences that has also been reported previously with static faces with neutral expression, with static and dynamic emotionally expressive stimuli, and also within dyadic social interactions (Blais et al., 2008; Haensel et al., 2020; Jack et al., 2009; Senju, Vernetti, Kikuchi, Akechi, Hasegawa, Johnson, 2013). Contrary to predictions, however, British participants did not show greater eye looking than Japanese individuals in the static or dynamic-neutral conditions. Consequently, the more distinct triangular scanning pattern of the eyes and mouth in WC compared to EA populations (Blais et al., 2008; Kelly et al., 2010; Kelly, Liu et al., 2011; Rodger et al., 2010) could not be replicated. In contrast to British participants, Japanese individuals were also expected to engage in both greater eye looking in the dynamic-expressive condition, as well as increased central face (nose) scanning for faces with neutral expressions (static and dynamic-neutral), but these predictions were not supported by the current results. Instead, Japanese individuals exhibited more central face (nose) scanning than the British in both the dynamic-neutral and dynamic-expressive conditions, although it should be noted that proportional nose scanning time was small in both cultural groups (see Fig. 3). The predictions for the current study were based on the possibility that the diverging scanning patterns reported in previous studies may have resulted from using neutral versus emotionally expressive face stimuli, but the present findings suggest that other factors also modulated scanning patterns in previous studies. For instance, whereas the current study involved a free-viewing paradigm, which is suitable for infant populations who cannot comply with verbal task instructions, eye movement behaviours observed in previous studies may arise from the underlying (often verbally instructed) experimental tasks. Eye scanning could have reflected a beneficial task-relevant strategy for British individuals during face recognition as previously reported (Blais et al., 2008), and for Japanese individuals during emotion categorisation given findings that East Asians represent the intensity of emotional expressions with movements of the eyes more than Western Caucasians (Jack et al., 2009, 2012). However, Senju, Vernetti, Kikuchi, Akechi, Hasegawa, Johnson (2013) also adopted a free-viewing paradigm and in contrast to the present findings found increased eye scanning for emotionally expressive faces in Japanese participants, suggesting that task differences cannot fully account for the differences in observed eye scanning between previous and current studies. An additional methodological factor that may influence face fixation patterns concerns stimulus differences in mouth movements. Unlike earlier studies (including Senju, Vernetti, Kikuchi, Akechi, Hasegawa, Johnson, 2013), the present dynamic displays additionally showed actors speaking unintelligible syllables. It has been shown that increased noise levels during speech can result in greater attention to the mouth region (Vatikiotis-Bateson, Eigsti, Yano, & Munhall, 1998), likely as a compensatory strategy for language understanding. Further supportive evidence comes from Thompson and Malloy (2004) who found that older adults (mean 71.5 years) scanned the mouth region significantly more than younger adults (mean 23.4 years) at the expense of the eye region. It is possible that the unintelligible speech in the present dynamic-neutral and dynamic-expressive conditions differentially modulated scanning behaviour in the two cultural groups. British participants may have engaged in greater mouth looking to decode unintelligible speech, whereas Japanese participants could have increasingly focused on the nose region to extract visual information from the mouth parafoveally. Consistent with this interpretation, both WCs and EAs have previously been shown to fixate the eyes and also the mouth (of a static face) when visual information was highly constrained (2° or 5°), but a shift toward a central fixation bias was observed for EAs only when both the eyes and mouth were visible at 8° (Caldara, Zhou, & Miellet, 2010). Overall, further systematic investigations for the effects of stimulus and task manipulations on face scanning will be required, as well as the interaction between these factors and the effects for cultural differences.

In addition to cultural effects, age-related differences on face scanning were also found, independent of cultural differences. Ten-month-old infants showed higher proportional fixation times on the eye region than 16-month-olds and adults. Conversely, mouth looking tended to be highest in the 16-month group across all stimulus types, although proportional mouth scanning times were very small for static faces irrespective of age group (see Fig. 4). As outlined in the introduction, this shift from eye to mouth looking between 10 and 16 months of age could reflect adaptive mechanisms for social learning through eye contact and gaze following at 10 months (Csibra & Gergely, 2006; Kleinke, 1986; Scaife & Bruner, 1975; Senju & Csibra, 2008), to language learning at 16 months (Hillairet de Boisferon et al., 2018). This would also be in line with the current findings suggesting higher proportional mouth scanning times in the 16-month group for dynamic faces, which showed actors articulating syllables (see Fig. 4). Given that the mouth region was moving for dynamic faces, low-level saliency could have also captured the visual attention of 16-month-olds. However, this unlikely accounts as a single explanation since younger infants are typically less able to disengage from visually salient regions (Johnson, Posner, & Rothbart, 1991), but the present 10-month group exhibited more eye scanning even in the dynamic-neutral condition for which the eyes were relatively motionless. Additional evidence supportive of a link between language learning and face scanning comes from studies demonstrating increased mouth scanning in bilingual compared to monolingual infants (Pons, Bosch, & Lewkowicz, 2015), and in infants who were presented with faces speaking a non-native compared to a native language (Lewkowicz & Hansen-Tift, 2012). In addition, an association between amount of mouth scanning and expressive language skills has been found in infants (Tenenbaum, Sobel, Sheinkopf, Malle, & Morgan, 2015; Tsang, Atagi, & Johnson, 2018), supporting the idea that a looking bias toward the mouth may reflect an adaptive mechanism for language learning. Furthermore, while 10-month-olds consistently showed more eye and less mouth scanning than 16-month-olds across all three face stimulus types, age comparisons with adults differed slightly between stimulus types. Compared to 16-month-olds, for instance, adults showed significantly less mouth scanning for dynamic-expressive faces but no differences were found for dynamic-neutral faces. This could indicate more adaptive scanning strategies across the face to flexibly and dynamically extract social and language cues, with such greater face exploration also eliciting the observed higher proportional nose looking time compared to the two infant groups.

Crucially, these age-related differences did not interact with cultural differences in our results. Possible explanations could involve methodological limitations such as data quality, which can differ between age and ethnicity groups (Blignaut & Wium, 2014; Saez de Urabain et al., 2015; Wass, Forssman, & Leppänen, 2014). In the current study, spatial offsets did not systematically differ between age or cultural groups (see Supplementary Materials), and the present ROIs were also sufficiently large to reduce the possibility of fixations being misclassified into the ‘wrong’ ROI. Additionally, the present data was pre-processed using GraFIX (Saez de Urabain et al., 2015), which was developed to code fixations given varying levels of data quality both within and between experimental groups. Given the two-step procedure of GraFIX, fixation detection is still possible when spatial precision is low (to an extent; see Supplementary Materials for the guidelines used for the current study) and automatic procedures would not have flagged a fixation. Although data quality undoubtedly remains a wider issue for eye tracking in developmental populations and across cultural groups, the current procedures attempted to minimise such effects and suggest that data quality alone unlikely accounts for the present findings.

It is possible that cultural differences in face scanning, at least those observed in the current study, were relatively established by 10 months of age. Consistent with findings showing cultural influences on face scanning at 7 months when using static images of emotionally expressive faces (Geangu et al., 2016), cultural differences may have emerged prior to 10 months of age. Studies have demonstrated that young infants tend to orient to and fixate highly salient regions (Frank, Vul, & Johnson, 2009), and it is possible that low-level motion could represent a very early source for cultural differences in face scanning that cuts through early visual acuity limitations. For instance, phonological differences between the English and Japanese languages could affect the degree of articulation and therefore low-level motion within the mouth region. With greater visual information located in the mouth area in the English compared to Japanese language (Sekiyama & Tohkura, 1993), this could drive British infants to fixate the mouth more than Japanese infants. An additional source for early cultural differences in face scanning may relate to caregivers’ facial expressivity (cf., Geangu et al., 2016). East Asian mothers reportedly show less facial emotional expressivity than Western Caucasian mothers (Fogel, Toda, & Kawai, 1988), and the locations of visually informative regions for emotional expressions may therefore differ between cultures. As infants acquire more visual experience with the caregiver’s face, they may learn to attend to the visually informative regions. Such cultural learning via the caregiver suggests a developmental mechanism that would also be consistent with two studies that highlight the significant role of early familial experience in social development. For instance, Senju et al. (2015) found that infants raised by blind parents attended less to dynamic eye gaze while scoring typically on social communication measures. With respect to culture, Kelly, Jack et al. (2011) showed that while only 25–30 % of British Born Chinese (BBC) adults employed triangular scanning patterns of the eyes and mouth, informal interviews revealed that most BBCs were not much exposed to Western cultures until they started school. The role of the familial environment could therefore significantly impact the development of cultural differences in face scanning.

Future studies could consider including a much younger age group to contrast developmental trajectories for scanning behaviour between cultures within the first year of life. Developmental changes in the size of cultural differences may also be small in nature and thus particularly difficult to detect using unconstrained free-viewing paradigms that are typically necessary for infant populations, with other factors (e.g., language learning) playing a more prominent role in modulating face scanning strategies than cultural background. It is therefore also possible that age-related changes in cultural differences could be observed when employing face stimuli that could ‘induce’ certain cognitive processes in participants; for instance, dynamic, emotionally expressive displays without unintelligible speech (unlike the dynamic-expressive faces used in the present study, for which speech was unintelligible) may result in significantly less mouth scanning in Japanese but not British adults due to induced processing of facial expression of emotion (Haensel et al., 2020; Jack et al., 2012; Senju, Vernetti, Kikuchi, Akechi, Hasegawa, Johnson, 2013), which in turn may reveal age-related changes in cultural differences. In addition, studies on early adoptees (e.g., East Asian individuals raised entirely in a Western culture, or vice versa) would help delineate the role of parental behaviour, infant characteristics, and the timing of exposure to parents. Furthermore, ‘gene-culture co-evolution theory’ explains how genetic and cultural variations may have interdependently emerged (Beja-Pereira et al., 2003; Futuyma, 2017). Another possible direction for future research thus concerns the study of gene-culture interactions, whereby cultural influences interact with genetic predispositions to change phenotypical expression, such as potentially parental behaviour or infant visual attention. Given the current criticism on the candidate gene approach for cross-cultural psychology and psychiatry (Hamer & Sirota, 2000), future studies will benefit from a combination of refined methodological (Duncan, Ostacher, & Ballon, 2019) and theoretical (Finlay, Hinz, & Darlington, 2011; Karmiloff-Smith, 2006) approaches.

The initial analysis also included ethnicity of face stimuli as a factor in order to consider previous evidence that demonstrated modulating effects on face scanning (Fu et al., 2012; Liu et al., 2011; Wheeler et al., 2011; Xiao et al., 2013). However, such an ethnicity effect was not observed in the current study, similar to some earlier findings (Blais et al., 2008; Geangu et al., 2016; Senju, Vernetti, Kikuchi, Akechi, Hasegawa, Johnson, 2013). It is possible that face ethnicity effects are small in nature, or that inconsistent findings may result from methodological differences between previous studies, including the adoption of different analysis approaches characterised by varying statistical sensitivities (Arizpe, Kravitz, Walsh, Yovel, & Baker, 2016).

The current study revealed that fixation locations during face viewing were modulated by cultural background, independent of age-related changes. The present findings thus point to an early emergence of cultural differences in face scanning within the first year of life. The lack of observable age-related increase of cultural differences further points to similar developmental trajectories in face scanning in British and Japanese individuals beyond the first year of life. Altogether, individuals adopted different strategies for extracting visual information from faces, in line with their culture and stage in development.

## Funding sources

This work was supported by the 10.13039/501100007155Medical Research Council (MR/K016806/1; G1100252), Wellcome Trust/Birkbeck Institutional Strategic Support Fund (204770/Z/16/Z), and 10.13039/501100001691Japan Society for the Promotion of Science (16H01880, 16H06301, 15H01846, 25245067). The funders had no role in study design, data analysis, interpretation of results, or writing of the report.

## CRediT authorship contribution statement

**Jennifer X. Haensel:** Conceptualization, Data curation, Funding acquisition, Investigation, Methodology, Project administration, Writing - original draft, Writing - review & editing, Formal analysis. **Mitsuhiko Ishikawa:** Investigation, Project administration, Writing - review & editing. **Shoji Itakura:** Funding acquisition, Supervision, Writing - review & editing. **Tim J. Smith:** Conceptualization, Formal analysis, Funding acquisition, Methodology, Supervision, Writing - review & editing. **Atsushi Senju:** Conceptualization, Formal analysis, Funding acquisition, Methodology, Supervision, Writing - review & editing.

## Declaration of Competing Interest

The authors report no declarations of interest.
