# Supplementary material for: Cultural influences on face scanning are consistent across infancy and adulthood
Source: Infant Behav Dev. 2020 Nov;61:101503. doi: 10.1016/j.infbeh.2020.101503 (PMC7768814; doi:10.1016/j.infbeh.2020.101503)
Supplement: Supplementary file 1 [file mmc1.docx]

**Supporting Information**

**Fixation coding**

GraFIX is a two-step fixation coding tool that first applies an automatic algorithm to detect fixations before allowing the user to manually flag, delete, or modify fixations (Saez de Urabain et al., 2015). The parameter settings for automatic fixation detection are reported in Table S1.

Table S1. Parameter settings for GraFIX.

| Parameter | Value |
| --- | --- |
| Fill missing data with opposite eye | Yes |
| Smoothing time (samples) | 30 |
| Smoothing space (samples) | 21 |
| Velocity threshold (°/second) | 20 |
| Maximum interpolation latency (milliseconds) | 150 |
| Maximum interpolation displacement (°) | 0.25 |
| Merge consecutive fixations with similar location (°) | 0.25 |
| Maximum root mean square per fixation (°) | 0.30 |
| Minimum fixation duration (milliseconds) | 100 |

The following guidelines were used for manual moderation of automatically coded fixations:

1. Fixations without a clear beginning and end are deleted.
2. A fixation will be coded if a clear beginning and end velocity spike can be observed and gaze data between the spikes remains relatively stable. A velocity spike exceeding the threshold – usually representing noisy data and not a saccade – typically prevents the fixation to be detected automatically. For highly noisy data, a fixation will be coded if the smoothed data forms separate gaze lines (as opposed to dot clusters due to highly variable data).
3. Two or more automatically coded fixations separated by very short periods of missing data are merged if the fixation positions remain constant.
4. Fixations will be merged if they are separated by microsaccades within the velocity threshold.
5. Very long fixations (> 5 seconds) characterised by a small, progressive drift in eye position are deleted since they likely indicate tiredness or boredom.

A second coder processed 20% of the data with excellent agreement for the number of detected fixations (ICC of 0.93, *p*< 0.001) and good agreement for the mean durations of fixations (ICC of 0.75, *p* < 0.001).

**Spatial offset**

Inward-turning spirals were presented between each task to measure spatial accuracy. The mean gaze coordinates of the final 20 data points before the disappearance of the spiral were obtained, and the distance to the true spiral location was calculated (*M* = 0.86º-1.16º; *SD =* 0.28º-1.11º). Spatial accuracy did not differ between groups (Group: *F*(1,136) = 3.15, *p*= 0.078; Age: *F*(2,136) = 0.37, *p*= 0.690; Group x Age: *F*(2,136) = 0.54, *p*= 0.584).

**Face scanning: Descriptive measures shown in Figure 3 (main text)**

| Type | ROI | Group | *Median* | *Lower 95% CI* | *Upper 95% CI* |
| --- | --- | --- | --- | --- | --- |
| Static | Eyes | British | 0.61 | 0.55 | 0.67 |
|  |  | Japanese | 0.70 | 0.57 | 0.73 |
|  | Nose | British | 0.11 | 0.08 | 0.14 |
|  |  | Japanese | 0.11 | 0.08 | 0.16 |
|  | Mouth | British | 0.10 | 0.06 | 0.13 |
|  |  | Japanese | 0.04 | 0.04 | 0.05 |
| Dynamic-neutral | Eyes | British | 0.32 | 0.23 | 0.36 |
|  |  | Japanese | 0.32 | 0.26 | 0.39 |
|  | Nose | British | 0.09 | 0.07 | 0.10 |
|  |  | Japanese | 0.15 | 0.12 | 0.18 |
|  | Mouth | British | 0.45 | 0.39 | 0.56 |
|  |  | Japanese | 0.36 | 0.28 | 0.42 |
| Dynamic-social | Eyes | British | 0.35 | 0.23 | 0.44 |
|  |  | Japanese | 0.40 | 0.30 | 0.51 |
|  | Nose | British | 0.09 | 0.07 | 0.13 |
|  |  | Japanese | 0.16 | 0.12 | 0.20 |
|  | Mouth | British | 0.36 | 0.28 | 0.42 |
|  |  | Japanese | 0.19 | 0.15 | 0.24 |

**Face scanning: Descriptive measures shown in Figure 4 (main text)**

| Type | ROI | Age | *Median* | *Lower 95% CI* | *Upper 95% CI* |
| --- | --- | --- | --- | --- | --- |
| Static | Eyes | 10 | 0.79 | 0.71 | 0.83 |
|  |  | 16 | 0.59 | 0.48 | 0.72 |
|  |  | Adults | 0.59 | 0.53 | 0.64 |
|  | Nose | 10 | 0.08 | 0.05 | 0.12 |
|  |  | 16 | 0.14 | 0.07 | 0.17 |
|  |  | Adults | 0.12 | 0.10 | 0.16 |
|  | Mouth | 10 | 0.02 | 0.09 | 0.04 |
|  |  | 16 | 0.10 | 0.05 | 0.18 |
|  |  | Adults | 0.07 | 0.06 | 0.10 |
| Dynamic-neutral | Eyes | 10 | 0.40 | 0.31 | 0.48 |
|  |  | 16 | 0.26 | 0.17 | 0.34 |
|  |  | Adults | 0.31 | 0.17 | 0.35 |
|  | Nose | 10 | 0.12 | 0.07 | 0.16 |
|  |  | 16 | 0.09 | 0.06 | 0.13 |
|  |  | Adults | 0.13 | 0.09 | 0.21 |
|  | Mouth | 10 | 0.30 | 0.23 | 0.37 |
|  |  | 16 | 0.51 | 0.43 | 0.62 |
|  |  | Adults | 0.45 | 0.36 | 0.54 |
| Dynamic-social | Eyes | 10 | 0.44 | 0.30 | 0.56 |
|  |  | 16 | 0.25 | 0.17 | 0.36 |
|  |  | Adults | 0.42 | 0.30 | 0.48 |
|  | Nose | 10 | 0.08 | 0.06 | 0.16 |
|  |  | 16 | 0.12 | 0.08 | 0.18 |
|  |  | Adults | 0.15 | 0.10 | 0.22 |
|  | Mouth | 10 | 0.26 | 0.17 | 0.38 |
|  |  | 16 | 0.42 | 0.27 | 0.61 |
|  |  | Adults | 0.23 | 0.17 | 0.28 |
